# Piezosurgery-Assisted Surgical Treatment in Impacted Canine Transmigration

**DOI:** 10.1155/2020/2687827

**Published:** 2020-04-29

**Authors:** Matheus Francisco Barros Rodrigues, Layla Louise de Amorim Rocha, Rodrigo da Franca Acioly, Cristofe Coelho Lopes da Rocha, Daniel do Carmo Carvalho

**Affiliations:** ^1^Dentistry Course, Faculdade Cathedral, Boa Vista 69307-053, Brazil; ^2^Department of Oral and Maxillofacial Surgery and Traumatology, Hospital Geral de Roraima, Boa Vista 69305-455, Brazil; ^3^Infrastructure Department, Instituto Federal, Boa Vista 69303-340, Brazil

## Abstract

Delayed eruption of a canine tooth may represent a possible impaction. If this is the case, a slight elevation in the palatal or vestibular mucosa is often observed. Cases of transmigration, where the unerupted tooth crosses the midline, are less frequent. This article reports on the piezosurgery-assisted surgical treatment of a transmigrated canine in the mentonian region. Treatment for this condition varies depending on the clinical characteristics, symptoms, and location of the dental element. The surgical treatment established for this case was satisfactory, and full recovery of the patient was achieved without complications such as an intense postoperative inflammatory response.

## 1. Introduction

In human dentition, mandibular and maxillary canines are teeth that have relevance both in aesthetics and occlusion [1–5]. A small manifestation such as a cyst or root fragment or mandibular trauma at an early age can cause dental deviation that may lead to impaction [3, 6]. Common precipitating factors are neighboring tooth lesions, infection, ectopic eruption, and interference with prosthesis [2].

Most impacted canines are clinically asymptomatic and do not cause pain or discomfort [3, 6, 7]. A high index of suspicion should exist for canine impaction in the case of delayed eruption and a slight elevation in the palatal or vestibular mucosa [7]. Vestibular positioning of impacted canines is usually associated with spatial problems, while palatal positioning of impacted canines is associated with path abnormalities [8].

Transmigration cases, where the unerupted tooth migrates and crosses the midline, are less frequent [5, 6, 9]. Studies show that the canines are the only teeth in the dental arch able to cross the midline [3]. This form of impaction occasionally occurs in the maxillary region; however, canine transmigration is more common in the mandible [10, 11]. In most cases, periapical radiographs are not sufficient to detect transmigrated canines, making a panoramic radiograph essential [6].

This article is aimed at reporting on the piezosurgery-assisted surgical treatment of a transmigrated canine in the mentonian region, showing the clinical and radiographic aspects as well as the established treatment. In addition, it reviews the main concepts of management and demonstrates a comparative and indicative analysis of the proposed treatments.

## 2. Case Report

**Figure 1:**
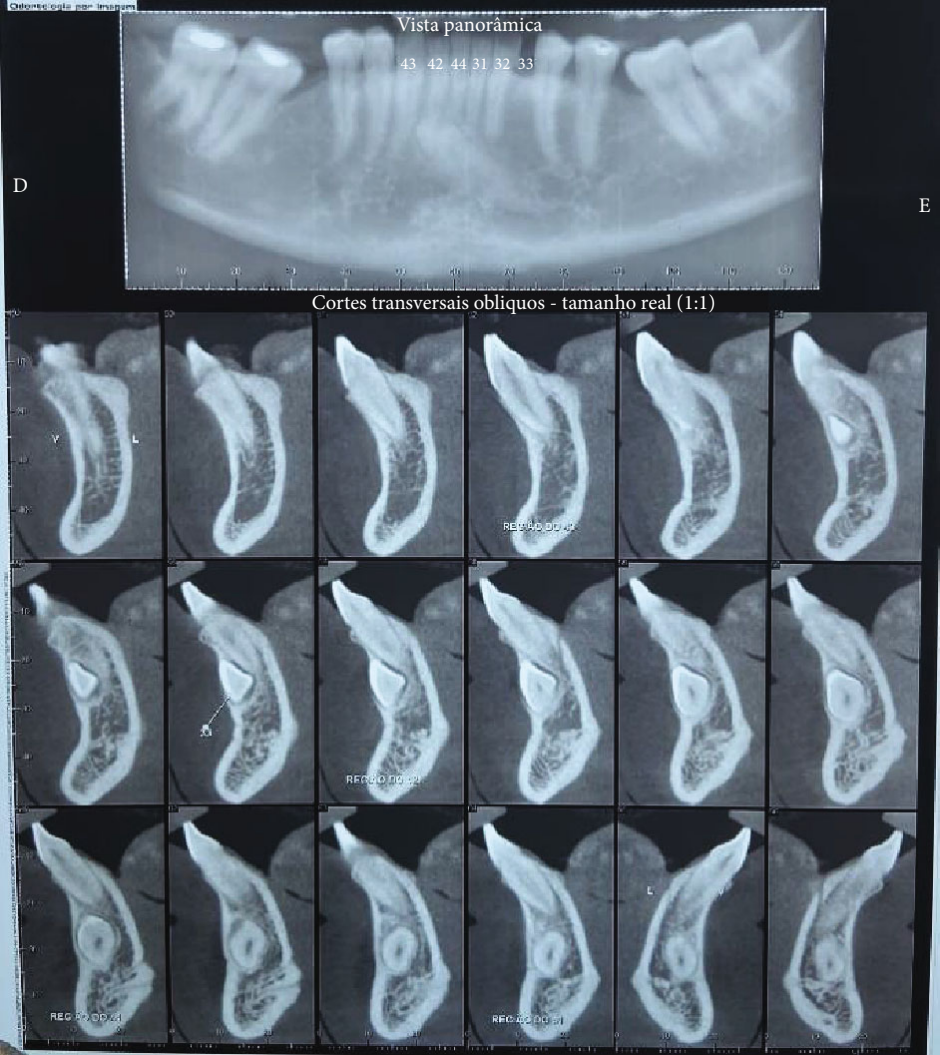
Computed tomography (CT) scan (panoramic view and oblique cross-sections).

A 33-year-old man presented to the dental office with the complaint of an absent lower canine on the left side. The patient had no other symptoms and was classified as ASA I. The clinical examination revealed the absence of dental element 33. In light of the above, a computed tomography (CT) scan was requested, and transmigration of the left lower canine in the midline in the mentonian region, in the horizontal and vestibular position, was diagnosed, as shown in Figure 1.

**Figure 2:**
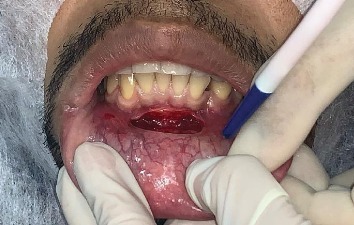
Horizontal incision 5 mm below the mucogingival line from canine to canine.

Due to the unfavorable position of the dental element, orthodontic treatment was contraindicated, establishing surgical extraction as the preferred mode of treatment [1, 7]. The patient underwent surgery under local anesthesia (articaine 4% 1 : 100,000) with bilateral blocks of the lower alveolar nerve, complemented by a vestibular canine-to-canine infiltration terminal. During surgery, a horizontal incision was made 5 mm below the mucogingival line extending from canine to canine, as shown in Figure 2. The tissue was then detached, allowing partial visualization of the impacted tooth.

**Figure 3:**
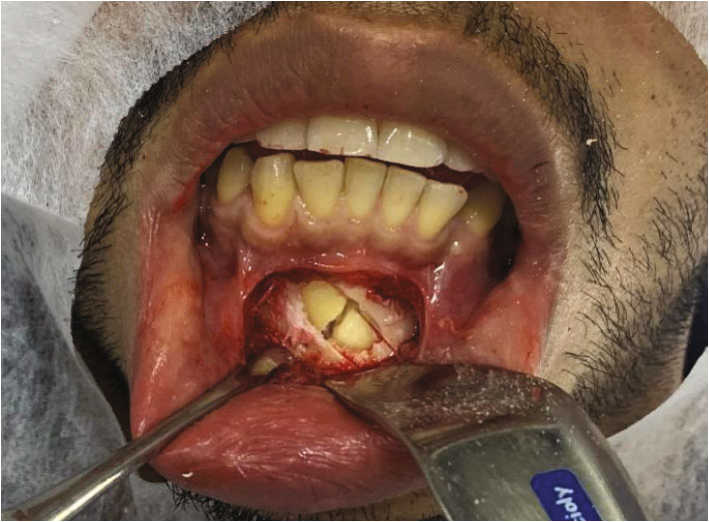
Osteotomy followed by piezosurgery-assisted odontosection.

The surrounding tissue was then separated from the tooth and odontosection was carried out using a piezoelectric apparatus in order to minimize the trauma, as can be seen in Figure 3.

**Figure 4:**
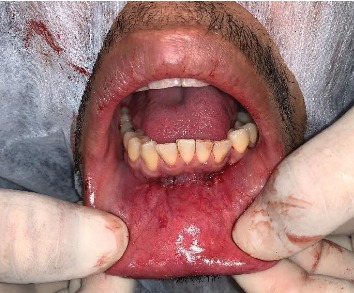
Suture in the deep and superficial plane.

After extracting the tooth, curettage and synthesis maneuver were performed in the internal musculature and in the superficial plane, both using an absorbable suture thread (Monocryl 5.0) as shown in Figure 4.

At the end of the surgical procedure, the patient received counselling regarding postoperative care. Antibiotic was prescribed (Amoxicillin 500 mg every 8 hours for 7 days) with an anti-inflammatory (Meloxicam 7.5 mg every 12 hours) and analgesic (Dipyrone Sodium 500 mg every 6 hours for three days). The patient underwent 5 laser therapy sessions during the recovery period and returned after 7 days to remove the sutures. At this presentation, there was no complaint of pain or paresthesia and no evidence of infectious symptoms.

## 3. Discussion

The teeth most affected by impaction are the third molars and permanent canines [12]. Surgeons should pay attention to the characteristics of impaction and select the appropriate treatment technique to appropriately manage each case on an individual basis [2].

Orthodontic traction and self-transplants were found in the literature as treatment options for impacted canines [6]. However, these are time-consuming and expensive treatments [2]. Important factors to consider when planning traction are patient age, root formation stage, tooth proximity to the site, tooth angulation between 0 and 15°, and the absence of root dilaceration [7].

Orthodontic traction of transmigrated teeth can be mechanically complex and may be contraindicated, depending on the angulation of these teeth [1, 7]. Following unsuccessful orthodontic treatment, surgical extraction is performed as a prophylactic measure against the formation of pathological lesions [1, 2].

Treatment success through the use of transplants depends on the degree of tooth development, as this procedure is more effective in cases where the impacted tooth has incomplete rhizogenesis. In addition, for the transplant to be viable, extensive ostectomy is necessary for full extraction of the impacted tooth and subsequent transplant placement in the dental arch [2].

Surgical treatment of an impacted element requires osteotomy and odontosection techniques that may damage the surrounding tissue [13]. However, piezosurgery-assisted surgery is useful in minimizing trauma [14], because the use of ordinary saws or high-speed drills applies pressure and a certain degree of heating to both the bone and the adjacent soft tissue. Use of the piezoelectric equipment results in comparatively minimum heating, therefore decreasing the risk of osteonecrosis by ensuring the vitality of the osteocytes [15].

## 4. Conclusions

It can been concluded that the surgical treatment strategy established for this case of transmigration and canine impaction was satisfactory and resulted in resolution of symptoms and a good clinical outcome. There was no intense inflammatory response or infectious condition in the postoperative period. The aid of piezosurgery assisted in minimizing trauma to soft and hard tissues and, therefore, reduced the risk of osteonecrosis in this case. Extraction of the dental element resulted in the prevention of future pathologies such as root resorptions and infections.
